# Supplementary material for: Procollagen I and III as Prognostic Markers in Patients Treated with Extracorporeal Membrane Oxygenation: A Prospective Observational Study
Source: J Clin Med. 2021 Aug 19;10(16):3686. doi: 10.3390/jcm10163686 (PMC8397027; doi:10.3390/jcm10163686)
Supplement: Supplementary file 1 [file jcm-10-03686-s001.zip › jcm-1289287-supplementary.pdf]

Supplement:

Supplemental Table S1. Mechanical ventilation and ECMO support during the course of study.

	**ICU Survivor (n = 13)**	**ICU Non-Survivor (n = 10)**	***p*-Value**
**V_T [mL/kg IBW]**			
day 0	3.2 ± 0.7	3.3 ± 0.5	0.6783
day 3	3.4 ± 0.7	3.4 ± 0.5	0.9506
day 5	4.6 ± 1.9	3.9 ± 1.6	0.3953
day 10	6.3 ± 2.2 [a,b]	4.4 ± 1.5	0.0664
**RR [breaths/min]**			
day 0	10 (10–12)	10 (10–11)	0.3788
day 3	10 (10–12)	10 (10–12)	0.6693
day 5	10 (10–16)	12 (10–16)	0.9339
day 10	15 (14–25) [a,b]	12 (10–14)	**0.0403**
**P_{plat} [cm H_2O]**			
day 0	24.2 ± 4.0	25.4 ± 4.9	0.5089
day 3	23.5 ± 4.1	27.0 ± 5.2	0.0813
day 5	23.4 ± 3.9	24.4 ± 4.3	0.5561
day 10	24.6 ± 3.7	25.1 ± 4.9	0.7880
**PEEP [cm H_2O]**			
day 0	17.5 ± 3.7	15.7 ± 4.4	0.3079
day 3	17.3 ± 3.9	16.3 ± 4.1	0.5502
day 5	16.3 ± 3.8	15.9 ± 4.1	0.8080
day 10	15.1 ± 3.7	16.1 ± 4.3	0.6028
**Driving pressure [cm H_2O]**			
day 0	6.0 (4.5–9.5)	6.5 (6.0–14.3)	0.1809
day 3	5.0 (4.0–10.0)	9.0 (6.0–16.0)	**0.0261**
day 5	7.0 (4.5–9.0)	7.0 (6.0–11.0)	0.4158
day 10	9.0 (7.0–11.0)	6.0 (5.0–15.0)	0.6240
**C_{rs} [mL/cm H_2O]**			
day 0	42.8 (32.4–53.8)	39.9 (20.0–47.0)	0.3434
day 3	50.0 (22.5–61.3)	26.8 (12.9–33.3)	**0.0220**
day 5	50.0 (26.4–58.2)	28.6 (18.8–41.7)	0.1139
day 10	47.0 (28.8–70.1)	41.7 (13.3–67.5)	0.4079
**ECMO flow [l/min]**			
day 0	4.1 ± 0.8	3.7 ± 0.8	0.2213
day 3	4.0 ± 0.7	3.6 ± 0.6	0.1741
day 5	3.4 ± 1.0	3.5 ± 0.7	0.8176
day 10	2.9 ± 0.8 [a,b]	3.1 ± 0.5	0.5721
**ECMO sweep gas flow [l/min]**			
day 0	4.0 (3.0–5.0)	3.5 (3.0–5.0)	0.7770
day 3	4.0 (3.5–5.0)	4.0 (3.8–5.0)	0.8753
day 5	4.0 (2.0–4.5)	4.0 (3.5–5.0)	0.3254
day 10	2.0 (1.0–3.0) [a]	4.0 (2.0–5.0)	0.1748
**Cumulative fluid balance [mL]**			
day 0	82 (−214–2609)	2135 (1065–5784)	0.1151
day 3	226 (−1701–6464)	16,134 (1978–25,073)	**0.0422**
day 5	−130 (−4194–3235)	15,505 (3887–27,574)	**0.0138**
day 10	−7020 (−9983–3815)	28,672 (191–38,013)	**0.0097**

Data are mean ± standard deviation or median (interquartile range). V_T, tidal volume; IBW, ideal body weight; RR, respiratory rate; P_{plat}, end-inspiratory plateau pressure; PEEP, positive end-expiratory pressure; C_{rs}, static compliance of the respiratory system, ECMO, extracorporeal membrane oxygenation. [a] denotes significant difference compared to day 0 ($p < 0.05$), [b] denotes significant difference compared to day 3 ($p < 0.05$).

V_T, P_{plat}, PEEP, ECMO flow and ECMO sweep gas flow did not differ between survivors and non-survivors. On day 10, RR in survivors was significantly higher compared to non-survivors (15 (14–25) vs. 12 (10–14) breaths/minute, $p = 0.0403$). There was no difference in driving pressure and C_{rs} between survivors and non-survivors at the time of ECMO initiation, day 5 and day 10. On day 3, driving pressure was lower (5.0 (4.0–10.0) vs. 9.0 (6.0–16.0) cm H_2O, $p = 0.0261$) and C_{rs} was higher (50.0 (22.5–61.3) vs. 26.8 (12.9–33.3) mL/cm H_2O, $p = 0.022$) in survivors compared to non-survivors. V_T and RR on day 10 were higher in survivors compared to the time of ECMO initiation (6.3 ± 2.2 vs. 3.2 ± 0.7 mL/kg IBW, $p < 0.0001$ and 15 (14–25) vs. 10 (10–12) breaths/minute, $p < 0.0001$) and day 3 (6.3 ± 2.2 vs. 3.4 ± 0.7 mL/kg IBW, $p = 0.0002$ and 15 (14–25) vs. 10 (10–12) breaths/minute, $p < 0.0001$). On day 10, ECMO flow was lower in survivors compared to the time of ECMO initiation (2.9 ± 0.8 vs. 4.1 ± 0.8 l/min, $p = 0.0035$) and day 3 (2.9 ± 0.8 vs. 4.0 ± 0.7 L/min, $p = 0.0058$). ECMO sweep gas flow in survivors was lower on day 10 than at the time of ECMO initiation (2.0 (1.0–3.0) vs. 4.0 (3.0–5.0) L/min, $p = 0.0204$).

At the time of ECMO initiation, non-survivors showed a non-significant trend for a higher cumulative fluid balance. The cumulative fluid balance in non-survivors was higher in non-survivors compared to survivors on day 3 (16,134 (1978–25,703) vs. 82 (−214–2609) mL, $p = 0.0422$), day 5 (15,505 (3887–27,574) vs. −130 (−4194–3235) mL, $p = 0.0138$) and day 10 (28,672 (191–38,013) vs. -7020 (−9983–3815) mL, $p = 0.0097$).

Supplemental Table S2. Inflammatory markers.

	Day 0	Day 3	Day 5	Day 10
**WBC [10^9/L]**				
ICU survivor (n = 13)	8.5 (7.7–14.1)	13.9 (7.3–29.0)	14.2 (9.2–21.5)	9.8 (7.5–13.4) [b]
ICU non-survivor (n = 10)	13.0 (7.8–26.0)	15.2 (10.5–18.7)	14.0 (8.4–22.8)	15.1 (10.4–27.2)
**CRP [mg/dL]**				
ICU survivor (n = 13)	260 (149–345)	159 (88–199)	105 (63–171) [a]	162 (45–214) [a]
ICU non-survivor (n = 10)	292 (163–357)	252 (103–356)	158 (80–347)	251 (117–274)
**Procalcitonin [ng/L]**				
ICU survivor (n = 13)	4.4 (0.7–29.5)	2.8 (0.6–11.6)	1.3 (0.3–2.8) [a]	1.2 (0.3–2.2) [a]
ICU non-survivor (n = 10)	20.2 (3.4–31.5)	4.2 (1.5–10.7)	2.2 (1.0–8.8) [a]	1.0 (0.4–3.1) [a]

Data are median (interquartile range). WBC, *white blood cell count*; CRP, *C-reactive protein*, [a] denotes significant difference compared to day 0 ($p < 0.05$), [b] denotes significant difference between survivors and non-survivors ($p < 0.05$).

As shown in Supplemental Table S2, CRP decreased between the time of ECMO initiation and day 5 (260 (149–345) vs. 105 (63–171) mg/dL, $p = 0.0035$) as well as day 10 (260 (149–345) vs. 162 (45–214) mg/dL, $p = 0.0493$) in survivors. PCT decreased between the time of ECMO initiation and day 5 or 10 in survivors (4.4 (0.7–29.5) vs. 1.3 (0.3–2.8) ng/mL, $p = 0.0426$ or 1.2 (0.3–2.2) ng/mL, $p = 0.0344$) and non-survivors (20.2 (3.4–31.5) vs. 2.2 (1.0–8.8) ng/mL, $p = 0.0411$ or 1.0 (0.4–3.1) ng/mL, $p = 0.0097$), respectively.

Supplemental Figure S1. Prediction of the logistic model.

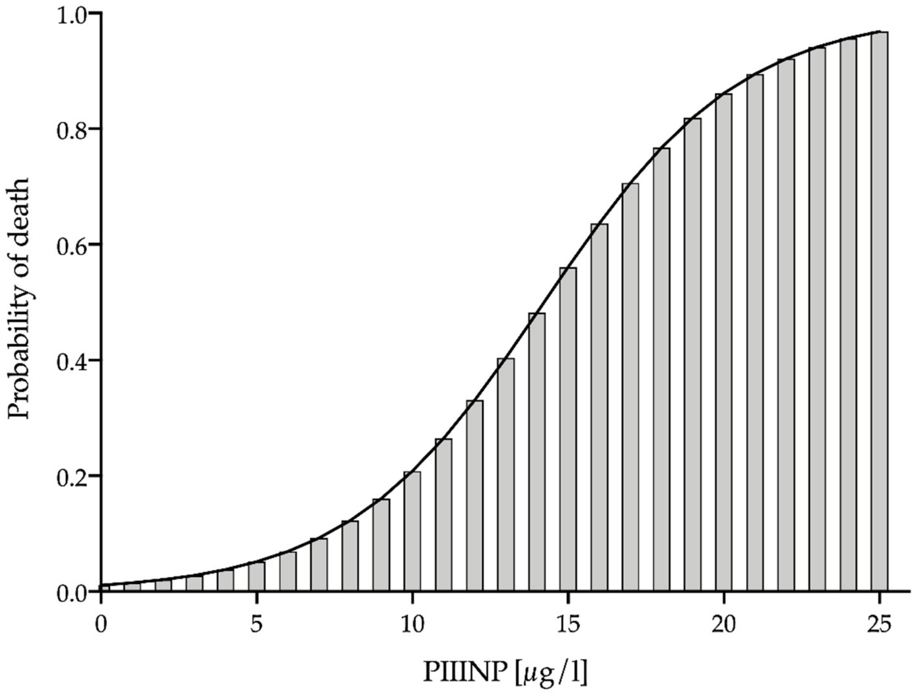

Supplemental Figure S2. Calibration curve.

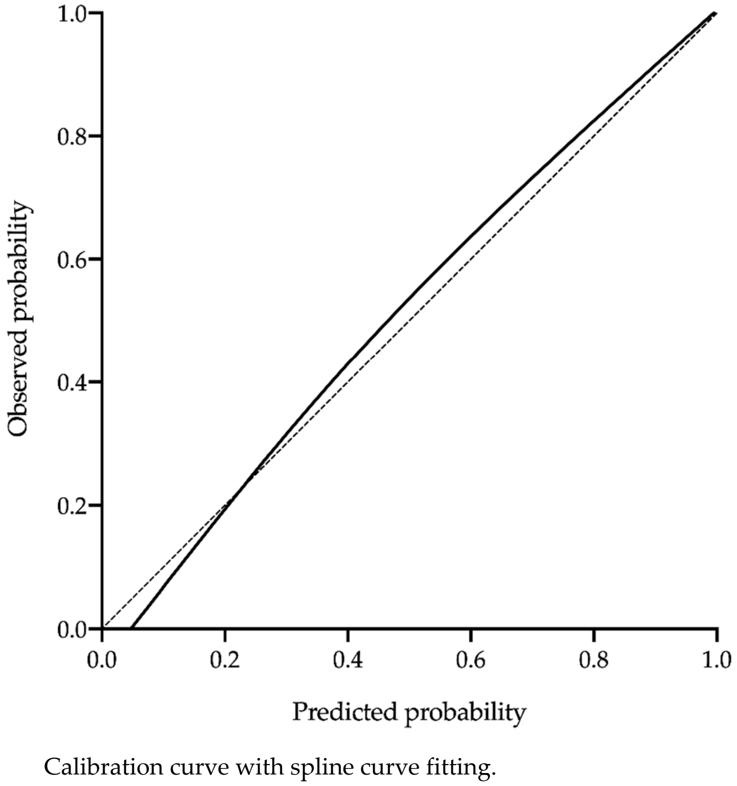

Calibration curve with spline curve fitting.

Supplemental Table S3: Contingency table of serum N-terminal procollagen III-peptide at the chosen cut-off.

	**ICU Survivor**	**ICU Non-Survivor**
**PIIINP > 12.8 μg/L**	1	9
**PIIINP < 12.8 μg/L**	12	1

Fisher's exact test significant ($p = 0.0001$) for the chosen cut-off
